# Bacterial diversity among the fruit bodies of ectomycorrhizal and saprophytic fungi and their corresponding hyphosphere soils

**DOI:** 10.1038/s41598-018-30120-6

**Published:** 2018-08-03

**Authors:** Yaping Liu, Qibiao Sun, Jing Li, Bin Lian

**Affiliations:** Jiangsu Key Laboratory for Microbes and Functional Genomics, College of Life Sciences, Nanjing Normal University, Nanjing, 210023 China

## Abstract

Macro-fungi play important roles in the soil elemental cycle in terrestrial ecosystems. Many researchers have focused on the interactions between mycorrhizal fungi and host plants, whilst comparatively few studies aim to characterise the relationships between macro-fungi and bacteria *in situ*. In this study, we detected endophytic bacteria within fruit bodies of ectomycorrhizal and saprophytic fungi (SAF) using high-throughput sequencing technology, as well as bacterial diversity in the corresponding hyphosphere soils below the fruit bodies. Bacteria such as *Helicobacter*, *Escherichia-Shigella*, and *Bacillus* were found to dominate within fruit bodies, indicating that they were crucial in the development of macro-fungi. The bacterial richness in the hyphosphere soils of ectomycorrhizal fungi (EcMF) was higher than that of SAF and significant difference in the composition of bacterial communities was observed. There were more Verrucomicrobia and Bacteroides in the hyphosphere soils of EcMF, and comparatively more Actinobacteria and Chloroflexi in the hyphosphere of SAF. The results indicated that the two types of macro-fungi can enrich, and shape the bacteria compatible with their respective ecological functions. This study will be beneficial to the further understanding of interactions between macro-fungi and relevant bacteria.

## Introduction

Macro-fungi, also known as mushrooms, are a type of chlorophyll-free heterotrophic organism^1^. Ectomycorrhizal fungi (EcMF) and saprophytic fungi (SAF) represent two major fungal guilds in terrestrial ecosystems and both play crucial roles in material conversion and elemental cycles^2–4^. EcMF are able to establish mutualistic interactions with host plants and form ectomycorrhizae in the natural environment^5^. They provide mineral elements for host plants by weathering minerals or decomposing organic matter^6,7^, or/and take-up mineral nutrients directly from soils to obtain valuable photosynthetic carbon in return^8,9^. The photosynthetic carbon is transferred to extramatrical mycelia and becomes a nutrient supply for underground heterotrophic organisms^10^. SAF are mainly responsible for decomposing litter and complex organic carbon in soils for nutrients^11,12^. Therefore, there are noteworthy differences in the ecological functions of the two types of macro-fungi in the terrestrial ecosystem.

The evolution of fungi in terrestrial ecosystems has exerted a strong impact on bacterial niche development^13^. Bacteria had developed competitive strategies for plant-derived substrates in their long-term evolution, and the utilisation of fungal-derived substrates has also led to different ecological strategies ranging from mutualist, endosymbiotic, and mycophagous bacteria^14^. In boreal forests, extramatrical mycelia of EcMF are important parts of underground biomass^15^. Compared to the root tips of plants, EcMF have a complex hyphal network and larger surface area which may provide a sufficient niche for relevant varied bacteria. Bacteria in ectomycorrhizosphere are not only influenced by the local soil environment, but strongly selected by particular fungal symbionts, namely, specific EcMF harboured distinct bacteria and ascomycete communities^16^. Tornberg and Olsson^17^ proposed that wood-decomposing fungi could influence bacterial community structure by using phospholipid fatty acid profiles to characterise bacterial communities. Furthermore, the bacteria may also be responsible for the changes in the structure of fungal communities demonstrated by the results from the study by Höppener-Ogawa^18^, who inoculated the genus *Collimonas* to soils and examined fungal diversity in soils and rhizosphere soils of arbuscular mycorrhizal fungi by PCR-DGGE. Therefore, fungal hyphae, to some extent, may affect the bacterial compositions of the underlying soils and a stable community structure in hyphosphere soils by mutual selection and adaptation processes.

Endophytic bacteria in fruit bodies have attracted more attention to the study of the microbiome of bacteria associated with macro-fungi. Pent and Bahram^19^ investigated and sequenced the endophytic bacteria from the fruit bodies of Agaricomycetes and found that both the soils, and the fungal species, contributed to the bacterial communities in fruit bodies. They hypothesised that the bacteria in fungal fruit bodies may be selected based on their symbiotic functions or environmental requirements. Benucci and Bonito^20^ also drew a similar conclusion, namely that fungal species, and their regional distribution may contribute to bacterial diversity associated with fruit bodies of Pezizales. Therefore, the bacteria living in the fruit bodies of macro-fungi may play an important role in the development of the fruit bodies.

Based on the aforementioned analysis, we proposed that the hyphae of EcMF and SAF are capable of maintaining, or regulating, some special bacterial populations in their fruit bodies and hyphosphere soils. Therefore, an experiment was conducted to reveal the ecological relationship between macro-fungi and bacteria *in situ*, which is conducive to understanding of their ecological roles.

## Results

### Molecular identification of the collected macro-fungi

**Figure 1:**
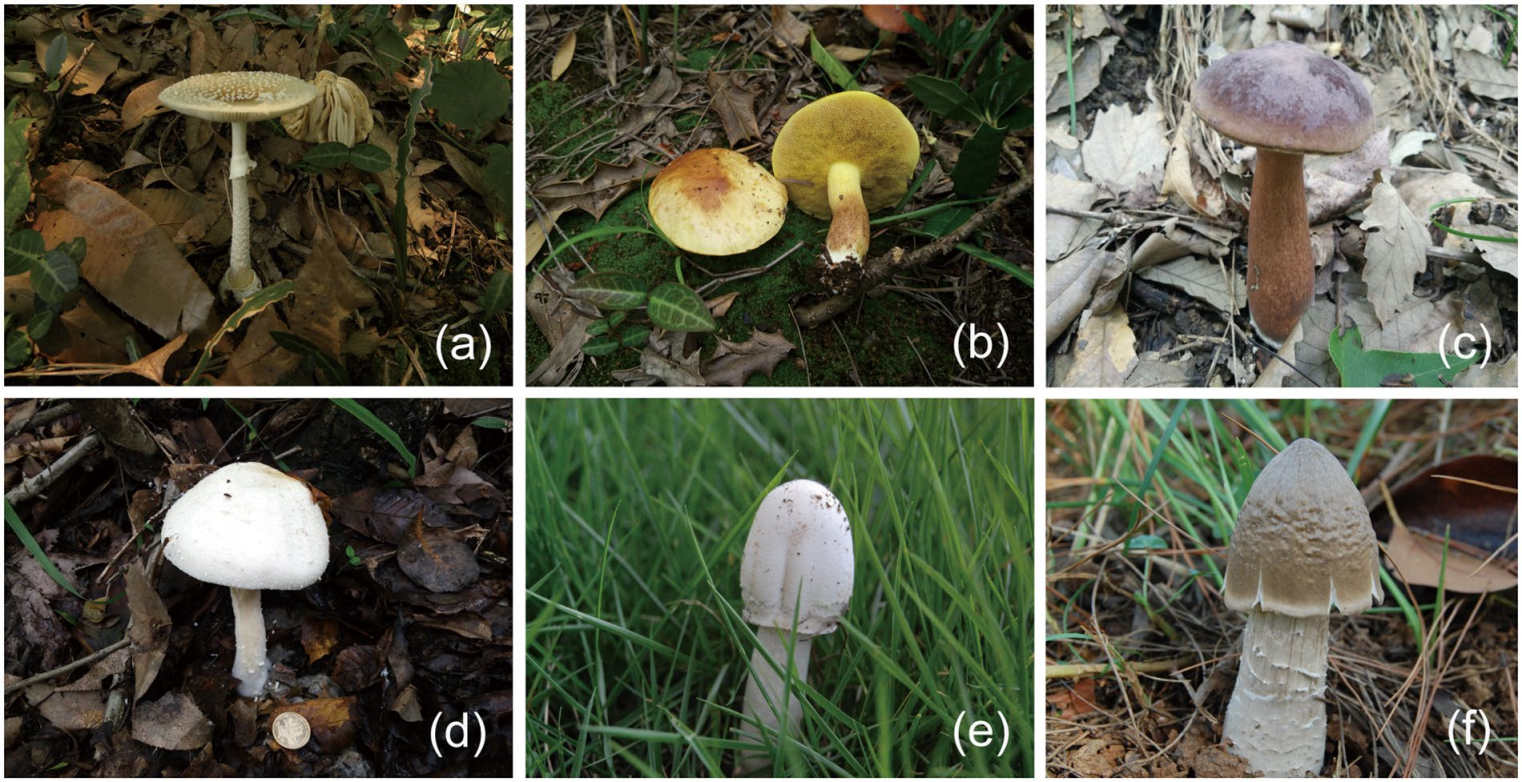
Photographs of the fruit bodies of *Amanita pantherina* (**a**), *Suillus placidus* (**b**), *Tylopilus felleus* (**c**), *Agaricus flocculosipes* (**d**), *Chlorophyllum molybdites* (**e**), and *Termitomyces albuminosus* (**f**).

Six species of macro-fungi collected were morphologically identified according to the Dictionary of the Fungi^21^, the results showed that the EcMF belonged to Amanitaceae and Boletaceae and SAF belonged to Agaricaceae and Tricholomataceae, respectively. Then a molecular method was employed to corroborate the fungi based on the ITS sequence and the BLAST results of the fungi were as follows (Table 1): the EcMF collected were: *Amanita pantherina* (Fig. 1a), *Suillus placidus* (Fig. 1b), and *Tylopilus felleus* (Fig. 1c), which are mainly symbiotic with *Pinus massoniana* and *Cyclobalanopsis glauca*, and the SAF collected were: *Agaricus flocculosipes* (Fig. 1d), *Chlorophyllum molybdites* (Fig. 1e), and *Termitomyces albuminosus* (Fig. 1f).

**Table 1:** The BLAST results of six species fungi.

Strain code	Referential taxon and accession numbers	Query coverage	Identity	E-value
LS08	*A. pantherina* (AB096044)	100%	100%	0.0
LS07	*S. placidus* (DQ407257)	98%	99%	0.0
LS90	*T. felleus* (JN182869)	100%	99%	0.0
LS095	*A. flocculosipes* (KP705076)	100%	99%	0.0
LS091	*C. molybdites* (KP012712)	97%	99%	0.0
LS092	*T. albuminosus* (KF302100)	99%	99%	0.0

Sequences of the ITS region of strains LS08, LS07, LS090, LS095, LS091, and LS092 were deposited in Genbank with accession numbers KR456156, KT381612, MG270070, MG270071, MG270072, and MG270073, respectively.

The endophytic bacteria within the fruit bodies of EcMF were regarded as bEMF, and the endophytic bacteria within the fruit bodies of *A. pantherina*, *S. placidus* and *T. felleus* were labelled as AP, SP, and TF, respectively; the endophytic bacteria within the fruit bodies of SAF were regarded as bSAF, and the endophytic bacteria within the fruit bodies of *A. flocculosipes*, *C. molybdites*, and *T. albuminosus* were labelled as AF, CM, and TA, respectively.

The bacteria of hyphosphere soils below the fruit bodies of EcMF were seen as EMFs, and the bacteria of hyphosphere soils below the fruit bodies of *A. pantherina*, *S. placidus* and *T. felleus* were labelled as APs, SPs, and TFs, respectively; the bacteria of hyphosphere soils below the fruit bodies of SAF were regarded as SAFs, and the bacteria of hyphosphere soils below the fruit bodies of *A. flocculosipes*, *C. molybdites*, and *T. albuminosus* were labelled as AFs, CMs, and TAs, respectively.

### Data analysis and bacterial diversity

**Figure 2:**
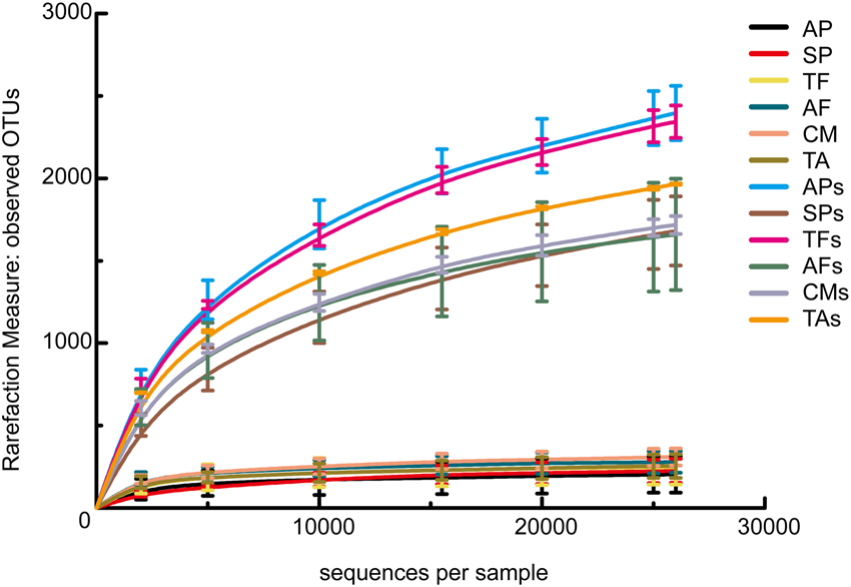
Rarefaction curves of observed OTUs (operational taxonomic units, OTUs) at 97% similarity. The average values of three replicates are shown for each sample including the standard error therein. AP, SP, and TF represented the endophytic bacteria of three species of ectomycorrhizal fungi, respectively, and APs, SPs, and TFs represented the bacteria of corresponding hyphosphere soils of ectomycorrhizal fungi, respectively. AF, CM, and TA represented the endophytic bacteria of three species of saprophytic fungi, respectively, and AFs, CMs, and TAs represented the bacteria of the corresponding hyphosphere soils of saprophytic fungi.

After merging and quality control, each experimental sample received more than 26,000 valid reads. The sample rarefaction curves of the operational taxonomic units (OTUs) (Fig. 2) showed that the sequencing depth covered nearly all bacterial communities in each of the samples and can be used for downstream analyses of bacterial diversity.

**Figure 3:**
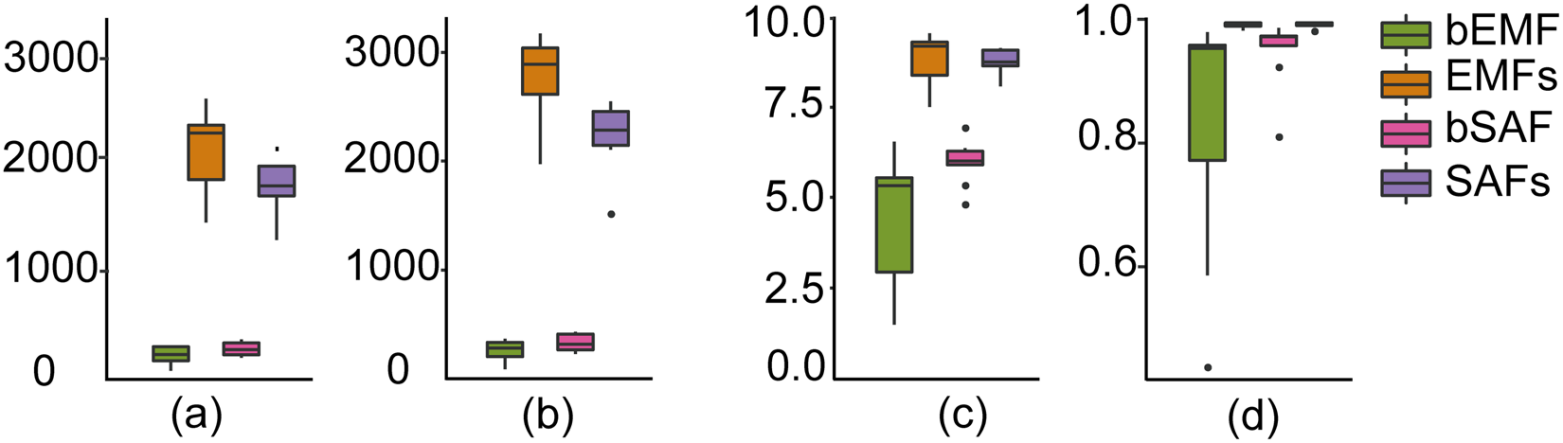
α-diversity indices of endophytic bacteria in two types of fruit bodies (bEMF and bSAF) and the bacterial in corresponding hyphosphere soils (EMFs and SAFs). (**a**) observed OTUs; (**b**) Chao1 index; (**c**) Shannon index; (**d**) Simpson index.

All samples were normalised to 26,000 reads for downstream analyses in QIIME. As for the alpha diversity of the bacterial communities, the richness of endophytic bacteria of EcMF (bEMF) was shown to be significantly lower than EMFs (*t* = −14.97, *p* = 0.00) from observed OTUs (Fig. 3a). A similar result was obtained in predicted OTUs by Chao1 (*t* = −18.96, *p* = 0.00) (Fig. 3b). Similarly, the same conclusions were drawn for the endophytic bacteria of SAF (bSAF).

In addition, the bacterial richness in hyphosphere soils below the fruit bodies of EcMF was greater than that of SAF. Conversely, the richness of endophytic bacteria in EcMF was lower than that of SAF. It can be seen from the Shannon and Simpson indices that the distribution of bacteria in the two types of fungal fruit bodies was more uniform than that in the corresponding soils (Fig. 3c,d).

**Figure 4:**
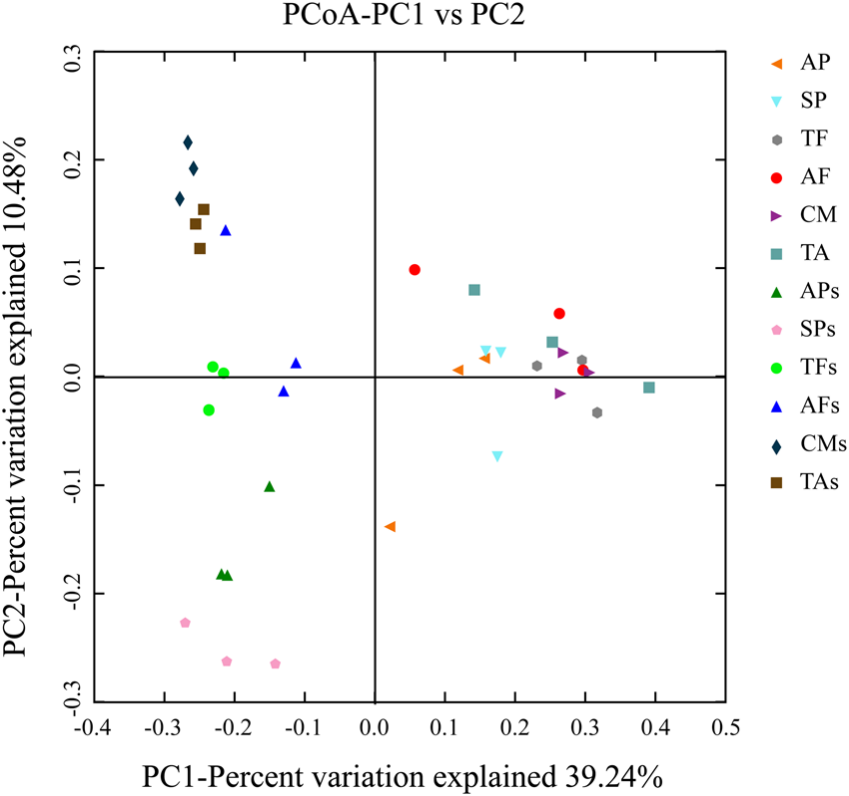
The principal coordinate analysis, namely, PCoA analysis, showed of the bacterial community composition on all analyzed samples at OTU level based on algorithm analysis by weighted_unifrac. The distance between points reflects the differences in bacterial community structure among samples.

PCoA analysis was mainly integrated the relative abundance and richness of species to explain the difference of bacterial communities in different groups. The PC1-axis was able to divide most of the bacteria in the corresponding soils into two parts (Fig. 4); however, it was unable to separate endophytic bacteria of fruit bodies entirely from two ecological types of macro-fungi. The PC2-axis was able to differentiate the bacterial community between fruit bodies and the corresponding soils. Although the hyphosphere soils were collected from the same sampling site, there were differences in the composition of bacteria between hyphosphere soils of different ecological types of fungi. However, the endophytic bacterial communities of different macro-fungi were similar (R = 0.80, *p* = 0.00, number of permutations = 999).

### The bacterial composition of each sample

**Figure 5:**
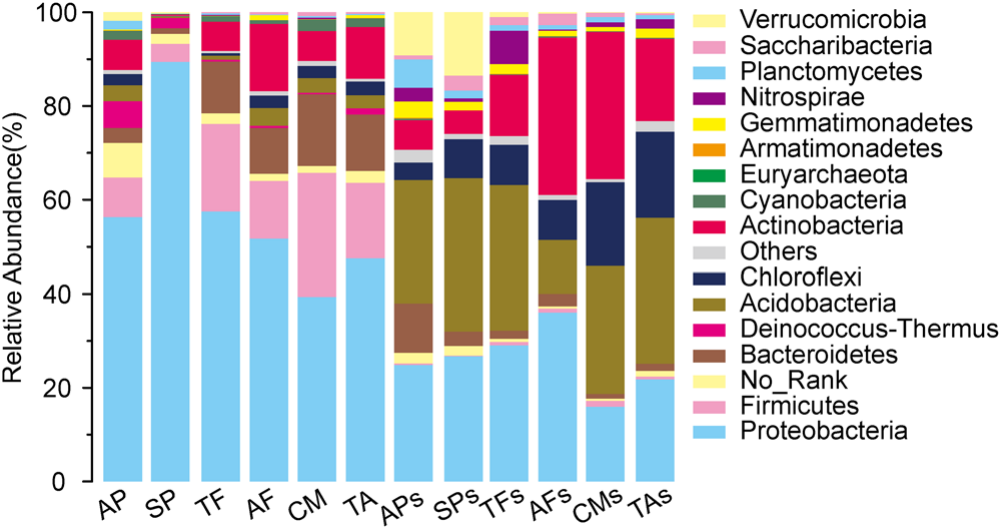
The comparison of bacterial composition based on the top 15 phyla of bacteria on all samples. Others, the remained phyla with lower relative abundance; No_Rank, no annotation information.

According to the annotation of OTUs, we found that endophytic bacteria within the fruit bodies or the bacteria in corresponding hyphosphere soils were mainly concentrated in Proteobacteria, Acidobacteria, Actinobacteria, Verrucomicrobia, Chloroflexi, Bacteroidetes, and Nitrospirae, which accounted for 67.09% to 94.58% of all test samples. The dominant endophytic bacteria groups in both the fruit bodies of EcMF and SAF were significantly different from the dominant bacteria in the corresponding soils. Proteobacteria dominated in both types of fruit bodies (39.36–89.48%), whilst it was not dominant in the corresponding soils (15.99–36.03%). Although the dominant bacteria at the level of phylum in the EMFs and SAFs were similar, their relative abundances differed (Fig. 5).

The compositions of bacteria at the level of genus were analysed and showed some differences between different groups (Table 2). The top five genera in bEMF: were *Enterobacter* (18.18 ± 0.25%), g_Enterobacteriaceae (12.11 ± 0.21%), *Burkholderia* (5.47 ± 0.13%), g_Xanthomonadaceae(3.99 ± 0.07%), and *Acinetobacter* (3.87 ± 0.058%); the top five genera in bSAF were *Helicobacter* (12.13 ± 0.13%), *Escherichia-Shigella* (4.86 ± 0.03%), *Bacteroides* (4.03 ± 0.03%), *Halomonas* (3.26 ± 0.03%), and *Bacillus* (3.14 ± 0.03%). The top five genera in EMFs were g_Acidobacteria (8.21 ± 0.04%), g_Chthoniobacterales (5.06 ± 0.05%), g_Acidobacteria (4.90 ± 0.04%), g_Acidobacteriaceae (3.67 ± 0.04%), and *g_* Acidobacteriales (2.77 ± 0.04%), and the top five genera in SAFs were g_Acidobacteria (10.34 ± 0.05%), g_RB41 (4.92 ± 0.04%), g_Chloroflexi (3.82 ± 0.02%), *Thermoleophilia* (3.00 ± 0.02%), and *Acidothermus* (2.50 ± 0.04%).

**Table 2:** Distribution of dominant bacteria from different sample groups based on the classification level of genus.

Phylum	Genus	bEMF	bSAF	EMFs	SAFs
Acidobacteria	g_Acidobacteriaceae	—	—	3.67 ± 0.042%	—
	g_Acidobacteriales	—	—	2.77 ± 0.041%	—
	g_RB41	—	—	8.21 ± 0.044%	4.92 ± 0.038%
	g_Acidobacteria	—	2.16 ± 0.022%	4.90 ± 0.039%	10.34 ± 0.048%
Actinobacteria	*Acidothermus*	—	—	—	2.50 ± 0.038%
	g_Micromonosporaceae	—	—	—	2.46 ± 0.027%
	g_Streptosporangiaceae	—	—	—	1.68 ± 0.028%
	*Thermoleophilia*	—	—	1.57 ± 0.010%	3.00 ± 0.021%
Bacteroidetes	*Bacteroides*	—	4.03 ± 0.040%	—	—
	*Prevotella 9*	1.04 ± 0.025%	2.80 ± 0.028%	—	—
Chloroflexia	*Roseiflexus*	—	—	—	1.86 ± 0.017%
	g_Chloroflexi	—	—	1.11 ± 0.011%	3.82 ± 0.018%
	g_Thermomicrobia	—	—	—	1.93 ± 0.014%
Deinococcus-Thermus	*Meiothermus*	2.59 ± 0.039%	—	—	—
Firmicutes	*Bacillus*	3.07 ± 0.031%	3.14 ± 0.031%	—	—
	*Exiguobacterium*	—	1.97 ± 0.020%	—	—
	*Faecalibacterium*	—	2.06 ± 0.021%	—	—
Gemmatimonadetes	g_Gemmatimonadaceae	—	—	1.89 ± 0.008%	—
Nitrospirae	g_Nitrospirales	—	—	2.42 ± 0.026%	—
Proteobacteria	*Bradyrhizobium*	—	—	1.69 ± 0.011%	1.86 ± 0.020%
	*Methylobacterium*	—	1.87 ± 0.019%	—	—
	*Sphingomonas*	1.98 ± 0.030%	1.44 ± 0.014%	1.83 ± 0.006%	1.46 ± 0.013%
	*Burkholderia*	5.47 ± 0.130%	—	1.41 ± 0.021%	—
	*Helicobacter*	2.28 ± 0.043%	12.13 ± 0.121%	—	—
	*Enterobacter*	18.18 ± 0.247%	1.25 ± 0.012%	—	—
	*Escherichia-Shigella*	1.97 ± 0.028%	4.86 ± 0.049%	—	—
	g_Enterobacteriaceae	12.11 ± 0.214%	1.68 ± 0.017%	—	—
	*Halomonas*	—	3.26 ± 0.033%	—	—
	*Acinetobacter*	3.87 ± 0.070%	1.46 ± 0.015%	—	—
	g_Xanthomonadaceae	3.99 ± 0.066%	—	—	—
Saccharibacteria	g_Saccharibacteria	—	—	1.86 ± 0.015%	1.10 ± 0.010%
Verrucomicrobia	g_Chthoniobacterales	—	—	5.06 ± 0.053%	—

“—”, the genus with relative abundance of less than 1%.

bEMF, the endophytic bacteria within the fruit bodies of EcMF; bSAF, the endophytic bacteria within the fruit bodies of SAF; EMFs, the bacteria of hyphosphere soils below the fruit bodies of EcMF; SAFs, the bacteria of hyphosphere soils below the fruit bodies of SAF.

### Intergroup comparison

**Figure 6:**
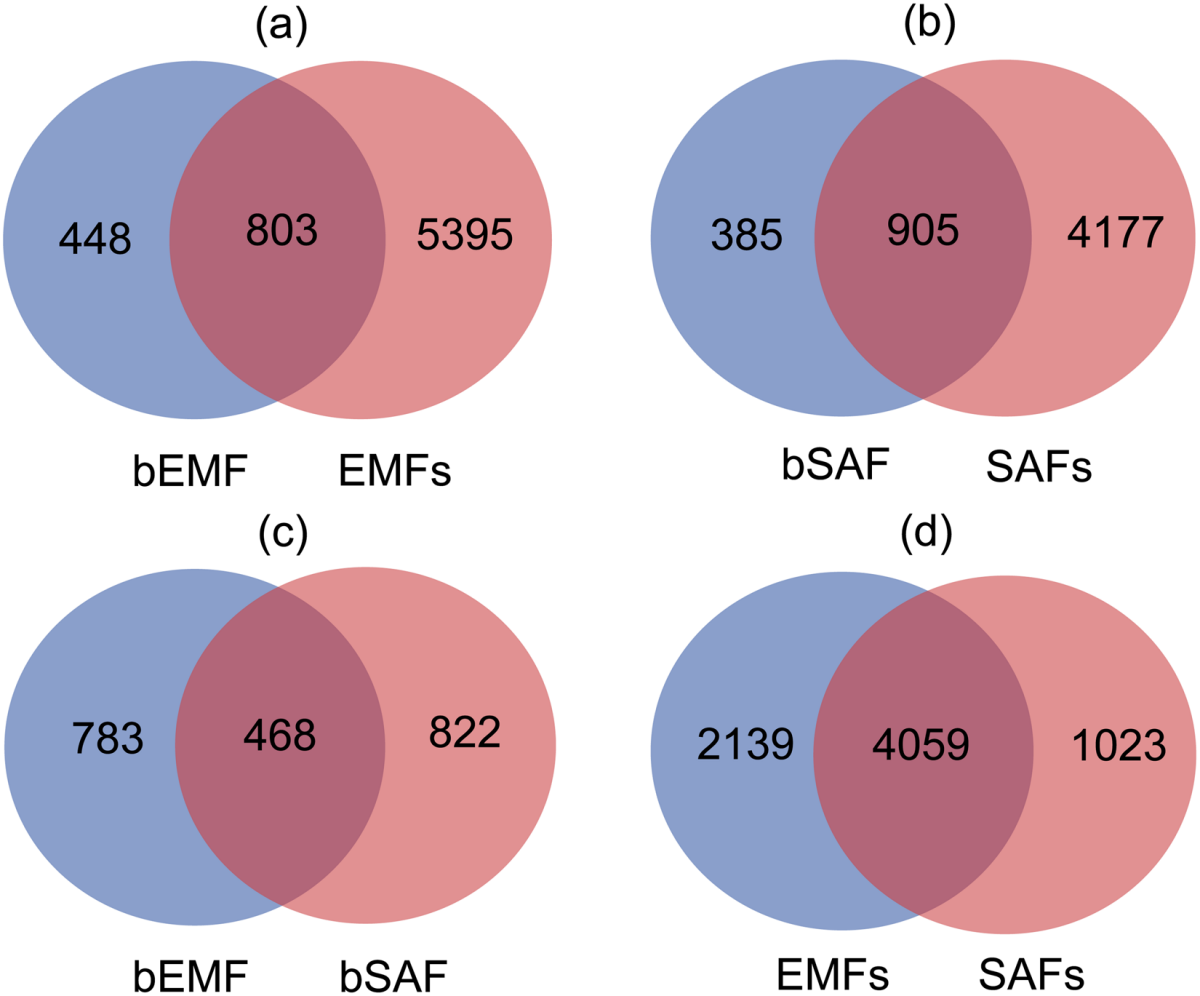
Venn diagram analysis for unique and shared OTUs among all the analyzed samples. (**a**) Comparison between bEMF and EMFs; (**b**) Comparison between bSAF and SAFs; (**c**) Comparison between bEMF and bSAF; (**d**) Comparison between EMFs and SAFs.

The comparison between groups showed that more than half OTUs in the fruit bodies can be detected in soils, which indicated the bacteria in the fruit bodies may mainly originate from the corresponding hyphosphere soils (Fig. 6a,b).

The common OTUs shared by the two types of fruit bodies accounted for less than 40% of the total number of OTUs (Fig. 6c), but the total reads of common OTUs were more than 75% of the total number of effective sequences. This indicated that the common bacteria occupied high relative abundances in the whole endophytic bacteria community within the two types of fruit bodies and that the common bacteria may play an important role in the life history of the fruit bodies. Moreover, a considerable part of the bacteria was common in both hyphosphere soils (Fig. 6d).

## Discussion

The bacteria inhabiting the macro-fungi are correlated with their hosts probably due to favourable growth environment and the selection of the fungi^22^. Rangel-Castro *et al*.^23^ analysed the growth media of *Cantharellus cibarius* by ^13^C-NMR and found exudation of trehalose and mannitol which may explain how millions of bacteria can reproduce inside long-lasting fruit bodies of chanterelles without damaging the hyphae thereof. The dominant bacteria may occupy the niche quickly and play a role in inhibiting the entry of other bacteria or pathogens, which has been confirmed in isolation experiments of endophytic bacteria in fruit bodies^24^.

The common endophytic bacteria of the two types of macro-fungi comprised the main bacteria groups, which was similar to the results found in a study by Dahm *et al*.^25^, who observed that the majority of bacteria derived from the fruiting bodies of EcMF were Gram-positive cocci. The similar physical environment and nutrients may account for the high abundance of common bacteria^23^. Furthermore, the high relative abundance of common bacteria may also be important for the growth of macro-fungi^22^. Tsukamoto *et al*.^26^ found that bacteria, such as *Acinetobacter* sp., *Bacillus pumilus*, and *Sphingobacterium multivorum*, isolated from wild Agaricales, are capable of detoxifying tolaasin produced by *Pseudomonas tolaasiithe*. Associated bacteria inhabit spores, the hyphal surface, and internal structures of arbuscular mycorrhizal fungi can promote growth of hyphae^24^, and accelerate the sporulation of arbuscular mycorrhizal fungi^27^. Similarily, bacteria associated with EcMF could play an important role in sporocarp formation^28^ and in promotion of mycorrhizal symbiosis^29–32^. Furthermore, some endophytic bacteria within plants exhibit a vertical transmission phenomenon in which endophytic bacteria are passed from parent to offspring through seeds conducive to the survival of the offspring^33^. In particular, fruit bodies of different fungal taxa create various specific conditions that filter certain bacteria from the surrounding bulk soil^34,35^. The endophytic bacteria within fruit bodies may be important to the growth of fruit bodies and the development of their spores.

Our results showed that the bacterial richness in EMFs was significantly higher than that in SAFs (*t* = 2.48, *p* = 0.03, Fig. 3a), which may be related to the fungus-derived carbon sources. Ectomycorrhizal symbiont can serve as a two-way channel to achieve transfer of the nutrition between plants and EcMF. EcMF provide a large number of mineral elements for plants and acquired valuable plant photosynthetic products in return^36,37^. The carbohydrates are an important carbon source of certain bacteria inhabiting the mycorrhizosphere^38^. So the hyphosphere soils of EcMF were able to support more types of bacteria.

**Figure 7:**
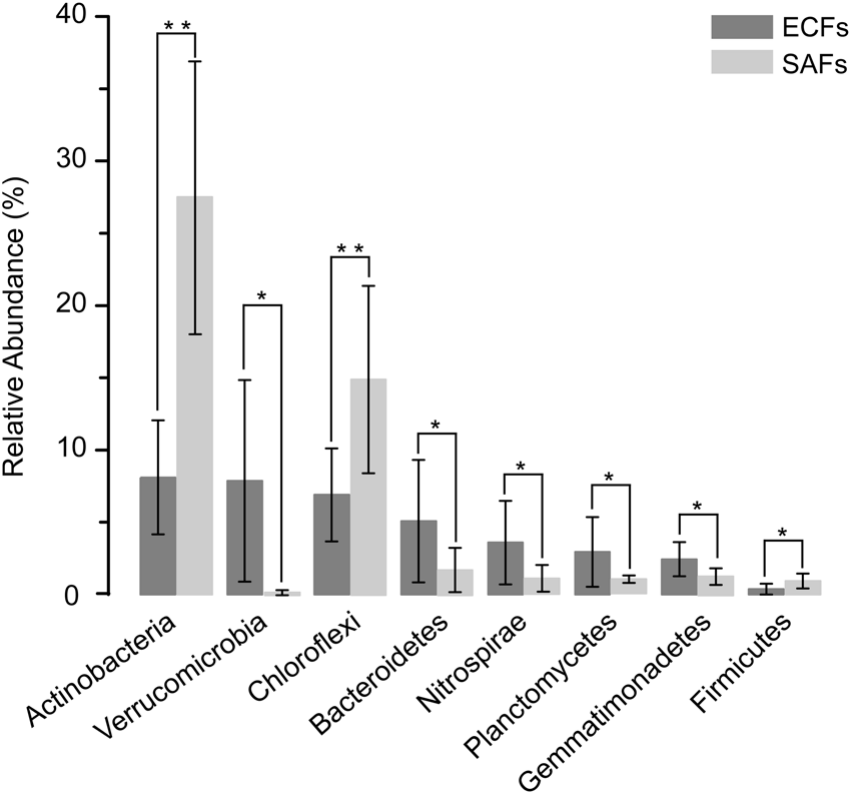
Differential analyses of bacteria in the hyphosphere soils of ectomycorrhizal fungi (EMFs) and saprophytic fungi (SAFs) at the level of phylum, and the phyla with significant differences between EMFs and SAFs were listed. *p*-values are derived from use of the *T-*test. *p < 0.05; **p < 0.01.

We analysed the bacteria with significant differences of the hyphosphere soils between two ecological types of macro-fungi based on the phylum level of classification (Fig. 7). The results showed that the growth of different fungal hyphae had a directional selection effect on the surrounding bacteria and tended to shape the microbial community to serve their respective ecological functions. It is interesting to note that Actinobacteria were dominant in SAFs, which may be associated with their producing rich antibiotics and inhibiting growth of pathogenic bacteria^39^, which were beneficial to the growth of hyphae of SAF and contribute to the formation of their fruit bodies. In contrast, EcMF were capable of secreting antibacterial substances which inhibit pathogenic microorganism, such as *Fusarium oxysporum*^40,41^. Thus the relative abundance of Actinobacteria in EMFs was markedly lower than that in SAFs. The microbial communities of the hyphosphere soils of EcMF were more conducive to the establishment of cooperative relationships between mycorrhizal fungi and specific plants. The measured pH of the soil samples showed the hyphosphere soils of the EcMF were generally acidic (Table 3). The acidic environment was beneficial to the release of insoluble mineral elements (such as K and P) and improved the efficiency with which the plant could use these mineral elements^42–44^. EcMF were likely to enrich the bacteria which were capable of producing acidic matter and weathering minerals^36,45^, and Taylor *et al*.^46^ found that EcMF secrete organic acids and decrease the pH of the surrounding soil to increase the content of mineral elements in the mycorrhizosphere. This might be a possible explanation to why Acidobacteria (*t* = 1.88, *p* = 0.09) and Bacteroides (*t* = 2.22, *p* = 0.05) were present in greater number in EMFs than that in SAF in this experiment. The EcMF also provided nitrogen to the plants^27^ which was consistent with the presence of a considerable portion of the Nitrospirae and Planctomycetes. Nitrospirae bacteria converted ammonium nitrogen into nitrate^28^ which was more easily absorbed and utilised by plants. These bacteria were involved in the cycle of nitrogen in the rhizosphere region. There were also some bacterial species with a special biological function in the hyphosphere soils of EcMF, mycorrhiza helper bacteria, which play an irreplaceable role in the formation of mycorrhizae, included *Bacillus* sp.^29^, *Pseudomonas* sp.^30^, and *Burkholderia* sp.^31^. The results of this study also proved that the relative abundance of these bacteria in the hyphosphere soils of EcMF was higher than that in SAF.

**Table 3:** The chemical properties of the hyphosphere soils below different types of macro-fungi.

Sample	pH	TOC	TN	Cu	Al	Ca	Fe	K	Mg	Mn	Ni	P	Zn
		mg/g		mg/kg									
APs	4.68 ± 0.15	4.61 ± 1.27	1.80 ± 0.11	0.40 ± 0.21	2.00 ± 0.33	378.17 ± 8.60	67.95 ± 16.55	54.43 ± 1.49	84.06 ± 7.46	15.17 ± 2.83	0.08 ± 0.10	0.41 ± 0.33	0.78 ± 0.55
SPs	6.00 ± 0.03	4.02 ± 2.91	1.53 ± 0.29	0.71 ± 0.41	1.43 ± 0.30	389.36 ± 4.71	50.14 ± 9.39	40.14 ± 7.33	57.28 ± 5.23	4.62 ± 1.20	0.00 ± 0.00	0.00 ± 0.00	0.64 ± 0.48
TFs	5.40 ± 0.07	4.71 ± 0.19	1.66 ± 0.05	0.84 ± 0.40	1.41 ± 0.41	367.31 ± 12.33	86.48 ± 23.19	88.39 ± 6.22	116.12 ± 3.26	7.04 ± 1.73	0.18 ± 0.17	0.00 ± 0.00	1.08 ± 0.77
AFs	4.61 ± 0.38	17.71 ± 7.60	2.99 ± 0.50	1.67 ± 0.92	11.28 ± 2.89	310.84 ± 46.64	122.02 ± 8.88	52.64 ± 28.27	16.95 ± 9.57	15.60 ± 3.29	0.00 ± 0.00	1.49 ± 0.99	6.85 ± 5.35
CMs	7.50 ± 0.14	10.62 ± 0.45	2.20 ± 0.01	1.53 ± 0.05	0.30 ± 0.04	389.72 ± 5.41	30.37 ± 0.14	104.81 ± 1.40	39.11 ± 0.52	5.32 ± 0.04	0.00 ± 0.00	0.55 ± 0.29	1.75 ± 0.05
TAs	7.69 ± 0.05	0.00 ± 0.00	1.14 ± 0.04	0.47 ± 0.02	0.38 ± 0.03	333.28 ± 3.37	25.15 ± 0.21	89.34 ± 1.61	159.12 ± 3.08	2.75 ± 0.03	0.00 ± 0.00	0.00 ± 0.00	0.00 ± 0.00

TOC, total organic carbon; TN, total nitrogen; Cu, exchangeable copper; Al, exchangeable aluminum; Ca, exchangeable calcium; Fe, exchangeable iron; K, exchangeable potassium; Mg, exchangeable magnesium; Mn, exchangeable manganese; P, exchangeable phosphorus; Zn, exchangeable zinc. Values are mean ± standard deviation.

## Methods

### The description of the sampling site and the collection of samples

Zijin Mountain is located in the east suburb of Nanjing city, Jiangsu Province, eastern China, and this location has a sub-tropical monsoon climate and has annual rainfall of 900–1000 mm. The vegetation types are mainly defoliation broadleaved forest with evergreen plants. Through a site investigation *in situ*, a suitable sampling site was found in the east of the foothills to Zijin Mountain (adjacent of the mountain itself), where the dominant trees are *Quercus* L. and *Pinus* Linn. Six species of macro-fungi were identified as potential research subjects. After morphological identification, the six species of fungi were divided into two ecological types of fungi, namely: EcMF, *A. pantherina* (LS08), *S. placidus* (LS07), and *T. felleus* (LS090); SAF, *A. flocculosipes* (LS095), *C. molybdites* (LS091), and *T. albuminosus* (LS092) referring to the source of the carbon, and the way in which they preliminary obtain carbon (Table 1). Three intact fruit bodies of each fungus were collected in July 2016, and a total of 18 fruit bodies were collected, placed on the ice and brought back to the laboratory.

Additionally, the hyphosphere soils below the corresponding fruit bodies (5 cm × 5 cm × 5 cm) were also collected and stored on ice in sterile Ziplock bags. Eighteen soil samples were collected in total. Each soil sample was blended and divided in two subsamples. One subsample was frozen at −80 °C for total bacterial DNA extraction and the others were air-dried and sieved (through a 1 mm square aperture sieve)) for the determination of specific soil properties. To measure the pH, 10 g dry soil samples were placed into a 100 mL Erlenmeyer flask and mixed with 25 mL water, shaken for 30 min, and then tested by pH meter SevenEasy (METTLER TOLEDO, Switzerland)^47^. Soil samples were treated with 1 mol/L hydrochloric acid and total organic carbon and total nitrogen was determined by vario EL III Element Analyzer^48^ (Elementar, Germany). Soil exchangeable cations and phosphorus were extracted using the ammonium bicarbonate-diethylenetriaminepentaacetic acid (AB-DTPA) multi-extractant method^49^, and the concentration of exchangeable cations and phosphorus were determined by Inductively Coupled Plasma Atomic Emission Spectrometer (LEEMAN LABS INC., USA).

### Identification of fungi

The modified pre-treatment of sporocarp was conducted with reference to Kumari *et al*.^22^. Briefly, the sporocarp surface was disinfected with 75% ethyl alcohol for 1 min, then as much of the inner tissues of the basidiocarps as possible were picked with a sterilised knife. The tissues linking pileus and stipe of EcMF were isolated from fruit bodies on solid Melin-Norkans medium (NaCl 0.025 g/L; (NH_4_)_2_HPO_4_ 0.25 g/L; KH_2_PO_4_ 0.5 g/L; FeCl_3_ 5 mg/L; CaCl_2_ 0.05 g/L; MgSO_4_∙7H_2_O 0.15 g/L; thiamine 0.1 g/L; glucose 10 g/L; casamino acids 1 g/L, malt 5 g/L, and agar 20 g/L in tap water^50^). Tissues linking pileus and stipe of SAF were isolated from fruit bodies on solid potato dextrose agar (potato 200 g/L; glucose 20 g/L; agar 20 g/L in tap water^51^). The purified fungal mycelia were selected to extract genomic DNA by Rapid Fungi Genomic DNA Isolation Kit (Sangon Biotech, China) according to the manufacturer’s instructions. The universal primers ITS1: 5′-TCCGTAGGTGAACCTGCGG-3′ and ITS4: 5′-TCCTCCGCTTATTGATATGC-3′ were employed to identify fungal taxa^52^.

### DNA extraction of endophytic bacteria and Illumina sequencing

Total bacterial DNA was extracted using a FastDNA^®^ Spin Kit for soils (MP Biomedicals, USA) according to the manufacturer’s instructions. Extracted DNA was diluted to 1 ng/μL with sterile deionised water according to the concentration. Primers Bakt_341F (5′-CCTACGGGNGGCWGCAG-3′) and Bakt_805R (5′-GACTACHVGGGTATCTAATCC-3′)^52^ (N = any base, W = A/T, H = A/C/T, and V = A/C/G) were used to amplify the V3-V4 region of the 16 S rDNA. Barcodes were added to the forward and reverse primers to attribute sequences to each sample. The program for V3-V4 region amplification was set so as to impose the following conditions: a lid temperature of 105 °C; initial denaturation for 30 s at 98 °C; annealing and Taq operation cycle repeated 30 times at 98 °C for 15 s, 58 °C for 15 s, and 72 °C for 1 min; followed by the final lengthening process at 72 °C for 1 min. All PCR reactions were performed with Phusion^®^ High-Fidelity PCR Master Mix. Agencourt AMPure XP 60 ml Kit (Beckman Coulter, USA) was used to purify PCR products. The purified PCR products were detected by Nanodrop (THERMO, USA) apparatus, to evaluate the quality of DNA. Then according to the concentration, target PCR products were mixed at an equimolar ratio and electrophoresed on 2% agarose gel and extracted using an AxyPrep™ DNA Gel Extraction Kit (AXYGEN SCIENTIFIC, USA). Sequencing libraries were quantifies using Library Quant Kit Illumina GA revised primer-SYBR Fast Universal kit according to the manufacturer’s recommendations. The library quality was assessed using the Qubit dsDNA HS Assay Kit (THERMO, USA) and Agilent Bioanalyzer 2100 system (AGILENT TECHNOLOGIES, USA). Finally, the library was sequenced on an Illumina Miseq platform by the Guhe Information Technology Co., Ltd, Hangzhou, China. The whole metagenomics dataset was submitted to Sequence Read Archive (SRA) of NCBI and the SRA accession is SRP128958.

### Data processing and statistical analyses

Raw sequencing data generated by Illumina were separated by samples according to barcode sequences. Raw reads were merged into a complete sequence in FLASH as supposed from valid reads^53^. The primer of the merged reads was moved by cutadapt^54^. Then, the high-quality clean reads thus obtained were used for OTU clustering by simultaneously removing singleton reads by using Uparse. Representative reads with the highest abundance in each OTU were selected for annotating information with SILVA^55^ by using the UCLUST algorithm^56^ in QIIME at 97% similarity. Finally, to ensure fair comparison between samples, all samples were normalised to the minimum number of effective reads and the normalised data were used for downstream analyses. Rarefaction curves and α-diversity indices of Chao1, Shannon, and Simpson were produced in QIIME. For β-diversity, an OTU level-based dissimilarity weighted_unifrac metric was used to measure the pair-wise community similarity between samples, and principal component analysis was used to visualise the distance matrix of all 36 samples in QIIME. A *T*-test was performed in SPSS 20 (IBM, USA).
